# Supplementary material for: ProteinShader: illustrative rendering of macromolecules
Source: BMC Struct Biol. 2009 Mar 30;9:19. doi: 10.1186/1472-6807-9-19 (PMC2672931; doi:10.1186/1472-6807-9-19)
Supplement: Additional file 1 — ProteinShader program without source code. This compressed file contains the complete ProteinShader program including associated libraries, but no source code. A README.txt file gives an overview of the ProteinShader distribution, and the index.html file in the help subdirectory has directions on getting started with the program as well as a set of tutorials. [file 1472-6807-9-19-S1.zip › ProteinShader-beta-0_9_4-binary/help/api/org/proteinshader/gui/package-use.html]

Uses of Package org.proteinshader.gui (ProteinShader API)


|  |  |  |
| --- | --- | --- |
| **Overview** \| **Package** \| Class \| **Use** \| **Tree** \| **Deprecated** \| **Index** \| **Help** | |  |
| PREV   NEXT | **FRAMES**    **NO FRAMES**     **All Classes** |  |


---


## **Uses of Package org.proteinshader.gui**

| Packages that use org.proteinshader.gui | |
| --- | --- |
| **org.proteinshader.gui** | Holds all of the Swing GUI components and their associated listeners, including class Renderer, which is registered as a listener for the GLCanvas object that is used a drawing surface. |
| **org.proteinshader.gui.components.controlpanel** | Holds the JPanels used in the right-side retractable control panel of the GUI. |
| **org.proteinshader.gui.components.menubar** | Holds the JMenus used in menu bar above the canvas. |
| **org.proteinshader.gui.listeners** | Holds the listener classes for the gui. |
| **org.proteinshader.gui.listeners.controlpanel** | Holds the listeners for components used in the JPanels of the right-side retractable control panel of the GUI. |
| **org.proteinshader.gui.listeners.menubar** | Holds the listeners for the items used in the JMenus of the menu bar above the canvas. |
| **org.proteinshader.gui.viewing** | Holds important helper classes that the Renderer uses to control the view: Camera, Rotation, Lighting, *etc*. |

Classes in org.proteinshader.gui used by org.proteinshader.gui

****Mediator****
  
          This interface specifies the methods for the single central mediator
object that allows communication between objects of the gui, the data
structure, and the renderer.

****ProteinShaderGUI****
  
          Provides the main GUI window for the ProteinShader program.

****Renderer****
  
          Knows how to render a protein image onto a GLCanvas.

Classes in org.proteinshader.gui used by org.proteinshader.gui.components.controlpanel

****Mediator****
  
          This interface specifies the methods for the single central mediator
object that allows communication between objects of the gui, the data
structure, and the renderer.

Classes in org.proteinshader.gui used by org.proteinshader.gui.components.menubar

****Mediator****
  
          This interface specifies the methods for the single central mediator
object that allows communication between objects of the gui, the data
structure, and the renderer.

Classes in org.proteinshader.gui used by org.proteinshader.gui.listeners

****Mediator****
  
          This interface specifies the methods for the single central mediator
object that allows communication between objects of the gui, the data
structure, and the renderer.

****Renderer****
  
          Knows how to render a protein image onto a GLCanvas.

Classes in org.proteinshader.gui used by org.proteinshader.gui.listeners.controlpanel

****Mediator****
  
          This interface specifies the methods for the single central mediator
object that allows communication between objects of the gui, the data
structure, and the renderer.

Classes in org.proteinshader.gui used by org.proteinshader.gui.listeners.menubar

****Mediator****
  
          This interface specifies the methods for the single central mediator
object that allows communication between objects of the gui, the data
structure, and the renderer.

Classes in org.proteinshader.gui used by org.proteinshader.gui.viewing

****Mediator****
  
          This interface specifies the methods for the single central mediator
object that allows communication between objects of the gui, the data
structure, and the renderer.

---


|  |  |  |
| --- | --- | --- |
| **Overview** \| **Package** \| Class \| **Use** \| **Tree** \| **Deprecated** \| **Index** \| **Help** | |  |
| PREV   NEXT | **FRAMES**    **NO FRAMES**     **All Classes** |  |


---

# *Copyright © 2007-2008*
